# Impact of COVID-related policies on gunshot wound assault hospitalizations in the United States: a statewide time series analysis

**DOI:** 10.1186/s40621-022-00412-7

**Published:** 2023-01-09

**Authors:** Paula D. Strassle, Jamie S. Ko, Madison Ponder, Anna María Nápoles, Alan C. Kinlaw, Sharon E. Schiro

**Affiliations:** 1 Division of Intramural Research, National Institute on Minority Health and Health Disparities, National Institutes of Health, Bethesda, MD USA; 2 Department of Epidemiology, Gillings School of Public Health, University of North Carolina at Chapel Hill, Chapel Hill, NC USA; 3 Division of Pharmaceutical Outcomes and Policy, University of North Carolina School of Pharmacy, Chapel Hill, NC USA; 4 Cecil G. Sheps Center for Health Services Research, University of North Carolina at Chapel Hill, Chapel Hill, NC USA; 5 Division of General, Acute Care, and Trauma Surgery, Department of Surgery, University of North Carolina at Chapel Hill, Chapel Hill, NC USA

**Keywords:** Gun violence, Stay-at-Home, Health disparities, Racial disparities

## Abstract

**Background:**

The CDC recently reported that firearm homicide rates in the United States increased in 2020, particularly among Black/African American individuals and men 25–44 years old. It is unclear whether firearm hospitalizations also increased, and more importantly, what impact the COVID-19 pandemic and COVID-related policies had. Using the North Carolina Trauma Registry, a statewide registry of trauma admissions to eighteen North Carolina hospitals, we calculated weekly GSW hospitalization rates from 1/2019 to 12/2020, overall and stratified by race-ethnicity, age, and sex. Interrupted time-series design and segmented linear regression were used to estimate changes in weekly hospitalization rates over time after (1) U.S. declaration of a public health emergency; (2) statewide Stay-at-Home order; (3) Stay-at-Home order lifted with restrictions (Phase 2: Safer-at-Home); and (4) further lifting of restrictions (Phase 2.5: Safer-at-Home). Non-GSW assault hospitalizations were used as a control to assess whether trends were observed across all assault hospitalizations or if effects were specific to gun violence.

**Findings:**

Overall, 47.3% (*n* = 3223) of assault hospitalizations were GSW. Among GSW hospitalizations, median age was 27 years old (interquartile range [IQR] 21–25), 86.2% were male, and 49.5% occurred after the U.S. declared a public health emergency. After the Stay-at-Home order was implemented, weekly GSW hospitalization rates began increasing substantially among Black/African American residents (weekly trend change = 0.775, 95% CI = 0.254 to 1.296), peaking at an average 15.6 hospitalizations per 1,000,000 residents. Weekly hospitalization rates declined after restrictions were lifted but remained elevated compared to pre-COVID levels in this group (average weekly rate 10.6 per 1,000,000 at the end of 2020 vs. 8.9 per 1,000,000 pre-pandemic). The Stay-at-Home order was also associated with increasing GSW hospitalization rates among males 25–44 years old (weekly trend change = 1.202, 95% CI = 0.631 to 1.773); rates also remained elevated among 25–44-year-old males after restrictions were lifted in 2020 (average weekly rate 10.1 vs. 7.9 per 1,000,000). Non-GSW hospitalization rates were relatively stable in 2020.

**Conclusions:**

The COVID-19 pandemic and statewide Stay-at-Home orders appeared to have placed Black/African American residents and men ages 25–44 at higher risk for GSW hospitalizations, exacerbating pre-existing disparities. Persistent gun violence disparities must be addressed.

**Supplementary Information:**

The online version contains supplementary material available at 10.1186/s40621-022-00412-7.

## Introduction

The Centers for Disease Control and Prevention (CDC) recently reported that the 2020 firearm homicide rates in the United States were the highest recorded since 1994, with the largest increases observed among Black/African American individuals, males, and adults 25–44 years old (Kegler et al. 2022). However, it is unclear whether gunshot wound (GSW) hospitalizations (fatal and nonfatal) have also increased during this time, and more importantly, whether COVID-related policies were associated with these changes. Our research group (Strassle et al. 2022) and others (Hatchimonji et al. 2020; Yeates et al. 2021; Abdallah et al. 2021) have previously reported that assault hospitalizations increased after Stay-at-Home orders in several states, but potential racial/ethnic, sex, and age disparities in the rates of GSW hospitalizations specifically during the pandemic have yet to be addressed. Thus, the purpose of this analysis was to assess changes in GSW assault hospitalizations over the first year of the pandemic among specific racial/ethnic, sex, and age groups, and the impact COVID-related policies had on hospitalization rates. Non-GSW assault hospitalizations were also assessed to determine if the potential impact of COVID-related policies applied to all assault mechanisms or only gun violence.

## Methods

We used data from the North Carolina Trauma Registry (NCTR), a statewide registry and cooperative effort between eighteen North Carolina hospitals, including all 17 trauma centers in the state (6 Level I, 3 Level II, and 8 Level III hospitals), and the North Carolina Office of Emergency Medical Services (Thomason 2008; Office of Emergency Medical Services). The NCTR has been in place since 1987 and collects near real-time information using standardized data definitions based off of the National Trauma Registry of the American College of Surgeons and designated chart abstractors. All hospitalizations where a patient is diagnosed with a traumatic injury (ICD-10-CM: S00-S99, T07, T14, T20-T28, T30-T32, T71, T79.A1-T79.A9), and is admitted to the hospital, taken to the operating room from the emergency department, transferred, or dies due to their injury are included. Unplanned readmissions within 30 days of the initial injury are also included.

For this study, we included all assault hospitalizations that occurred between January 1, 2019, and December 31, 2020. Both GSW and non-GSW assault hospitalizations were identified using the ICD-10-CM code framework from the National Center for Health Statistics and National Center for Injury Prevention and Control (Hedegaard et al. 2019). North Carolina population counts for 2019 were used to estimate the weekly hospitalization rates per 1,000,000 North Carolina residents. To account for variation between weekday and weekend admissions, we calculated the weekly rates of GSW and non-GSW assault hospitalizations between January 6, 2019, and December 26, 2020. Hospitalizations that occurred in partial weeks (i.e., January 1–5, 2019, and December 27–31, 2020) were excluded from our models to avoid introducing bias due to underestimating the total hospitalization rate for those weeks.

All patient demographics were abstracted from electronic medical records associated with the assault hospitalization by NCTR abstractors. Race and ethnicity were self-reported by the patient (or family member) if they were present and capable; otherwise, it was based on staff designation. Patients were categorized as non-Hispanic Black/African American, Hispanic/Latino, non-Hispanic White, and non-Hispanic other race; other race included individuals identified as American Indian, Asian, Pacific Islander, multiracial, or “other” race in their hospitalization record.

Differences in patient demographics and clinical characteristics among patients admitted for traumatic injuries between 2019 and 2020 were compared using standardized differences. An absolute difference > 0.20 was considered meaningful.

We then conducted a natural experiment using an interrupted time series design and segmented linear regression (Wagner et al. 2002; Taljaard et al. 2014). Interrupted times series is a powerful, quasi-experimental approach to evaluate longitudinal effects of interventions and policy changes that occur at a specific point in time. This approach is preferable to a more traditional “pre/post” test as it allows researchers to visualize both immediate (intercept) and gradual (slope) changes before and after the intervention, as opposed to just comparing the average rates before and after implementation (Wagner et al. 2002; Taljaard et al. 2014). These models can also incorporate multiple interventions within a single analysis, which is critical when assessing COVID-related policies. Using ordinary least squares, we ran race/ethnicity and sex/age-specific segmented linear regression models to estimate the trend in trauma hospitalization rates between each pair of COVID-related executive orders and policies.

The policies of interest included: U.S. declaration of a public health emergency (1/31/2020), statewide Stay-at-Home order (3/30/2020), partial lifting of Stay-at-Home restrictions (Phase 2: Safer-at-Home, 5/22/2020), and further lifting of restrictions (Phase 2.5: Safer-at-Home, 9/4/2020). Several statewide orders were not included in analyses because either the order made relatively small changes to existing orders (e.g., Phase 1 lifting of Stay-at-Home orders) or it occurred within several weeks of a prior order that we believed would be more salient (e.g., North Carolina declaring a state of emergency). A full list of executive orders and their effective dates are included in Additional file 1: Table S1.

To reduce error in our model, we used a transformed cosine periodic function to control for potential seasonal fluctuations in hospitalization rates.(Brookhart and Rothman 2008) To account for autocorrelation over time, we used Durbin–Watson tests (*α* = 0.05) to specify autoregressive parameters in our models for lags up to 60 weeks. Our models did not include parameters for level changes (i.e., intercept changes) to focus our analysis on an a priori-hypothesis that only gradual changes in injury hospitalization rates would be observed. Because no significant trend changes in rates were seen prior to the pandemic, the average weekly rate of hospitalizations for this time period was estimated by taking the mean of all estimated weekly rates prior to the U.S. declaration of a public health emergency. Post-policy slopes were calculated by summing the slope after the policy of interest (e.g., Stay-at-Home order) with the preceding slopes (e.g., pre-pandemic slope and slope after the U.S. declared a public health emergency) (Wagner et al. 2002; Taljaard et al. 2014). 95% confidence intervals were calculated using the approximate standard errors generated for each post-policy slope. We have used these methods previously (Strassle et al. 2022). Due to low rates, stratified models among women (all ages) and older men (≥ 65 years old) could not be performed.

All analyses were performed using SAS version 9.4 (SAS Inc., Cary, North Carolina). This study was deemed exempt by two Institutional Review Boards.

## Results

Overall, there were 6,818 assault injury hospitalizations between 2019 and 2020 in North Carolina; 47.1% were GSWs (*n* = 3223). Among GSW hospitalizations, median age was 27 (IQR 21–35), 86.2% were male, and 76.0% were Black/African American. There were no meaningful differences in the sociodemographics of GSW patients hospitalized in 2020, compared to 2019, Table 1. In 2020, 15.2% of GSW hospitalizations had confirmed (1.4%) or suspected (13.9%) COVID-19 infections. White GSW patients appeared slightly older (median age: 31 vs. 25–27), but minimal racial/ethnic differences were observed across injury severity score (median score: 9–10 for all), transfer status (22.0%–28.8%), emergency department length of stay (median stay: 2.0–2.7 h), and length of stay (median stay: 2–3 days for all), Additional file 1: Table S2. Minimal clinical differences were also observed across age among male GSW patients, Additional file 1: Table S3. Demographics and clinical characteristics for all assault hospitalizations can be found in Additional file 1: Table S4.

**Table 1:** Demographics and clinical characteristics among gunshot wound (GSW) assault hospitalizations included in the North Carolina Trauma Registry, 2019–2020, stratified by year

	2019		2020		Standardized difference^a^
Total, *N*	1374		1753		–
Age, years, med (IQR)	26	(20, 35)	27	(21, 34)	0.02
Age group, *n* (%)					
0–17	143	(9.8)	198	(11.3)	0.05
18–24	489	(33.4)	514	(29.3)	0.09
25–44	657	(44.8)	845	(48.2)	0.07
45–64	158	(10.8)	182	(10.4)	0.01
≥ 65	18	(1.2)	14	(0.8)	0.04
* Missing*	1		4		–
Male, *n* (%)	1272	(86.8)	1503	(85.7)	0.03
Race/ethnicity, *n* (%)					
American Indian	18	(1.2)	25	(1.4)	0.02
Asian	5	(0.3)	3	(0.2)	0.03
Black/African American	1100	(76.2)	1320	(75.8)	0.01
Hispanic/Latino	74	(5.1)	94	(5.4)	0.01
White	211	(14.6)	261	(15.0)	0.01
Other^b^	28	(1.9)	28	(1.6)	0.03
Multiracial	7	(0.5)	10	(0.6)	0.03
* Missing*	23		16		–
Primary payer, *n* (%)					
Any private insurance	226	(15.4)	218	(12.4)	0.09
Medicare/Medicaid only	373	(25.5)	538	(30.7)	0.12
Self-pay	795	(54.3)	909	(51.8)	0.05
Other^c^	69	(4.7)	89	(5.1)	0.02
Transferred to center, *n* (%)	346	(24.8)	432	(27.1)	0.05
ISS, med (IQR)	9	(1, 14)	9	(4, 16)	0.07
ED LOS, hours, med (IQR)	2.5	(1.0, 4.3)	2.5	(0.9, 4.3)	0.05
LOS, days, med (IQR)	2	(1, 6)	2	(1, 6)	0.04
ICU LOS^d^, days, med (IQR)	3	(1, 5)	3	(1, 5)	0.04
Discharge disposition, *n* (%)					
Routine/home	1,175	(83.2)	1,384	(82.4)	0.02
Long-term care^e^	75	(5.3)	81	(4.8)	0.02
Transferred^f^	44	(3.1)	57	(3.4)	0.02
Died	118	(8.4)	157	(9.4)	0.03
* Missing*^g^	54		78		–
COVID-19 infection, *n* (%)					
Confirmed	N/A		22	(1.3)	–
Suspected	N/A		245	(13.9)	–

med, median; IQR, interquartile range; ISS, injury severity score; ED, emergency department; LOS, length of stay; ICU, intensive care unit

^a^Absolute standardized difference (SD) comparing demographics and clinical characteristics between 2019 and 2020; an SD > 0.20 was considered meaningfully different

^b^Other race includes Other race and Hawaiian/Pacific Islander; race was collapsed due to small cell sizes

^c^Other insurance types include worker’s compensation, other government insurance, Champus, and not billed

^d^Among those admitted to ICU

^e^Long-term care includes: hospice, long-term care facility, nursing home, rehabilitation facility, skilled nursing facility (SNF)

^f^Transfers to: acute care facilities, burn center, mental health facility, other trauma centers, and transferred (unspecified)

^g^Includes individuals who left against medical advice

**Fig. 1:**
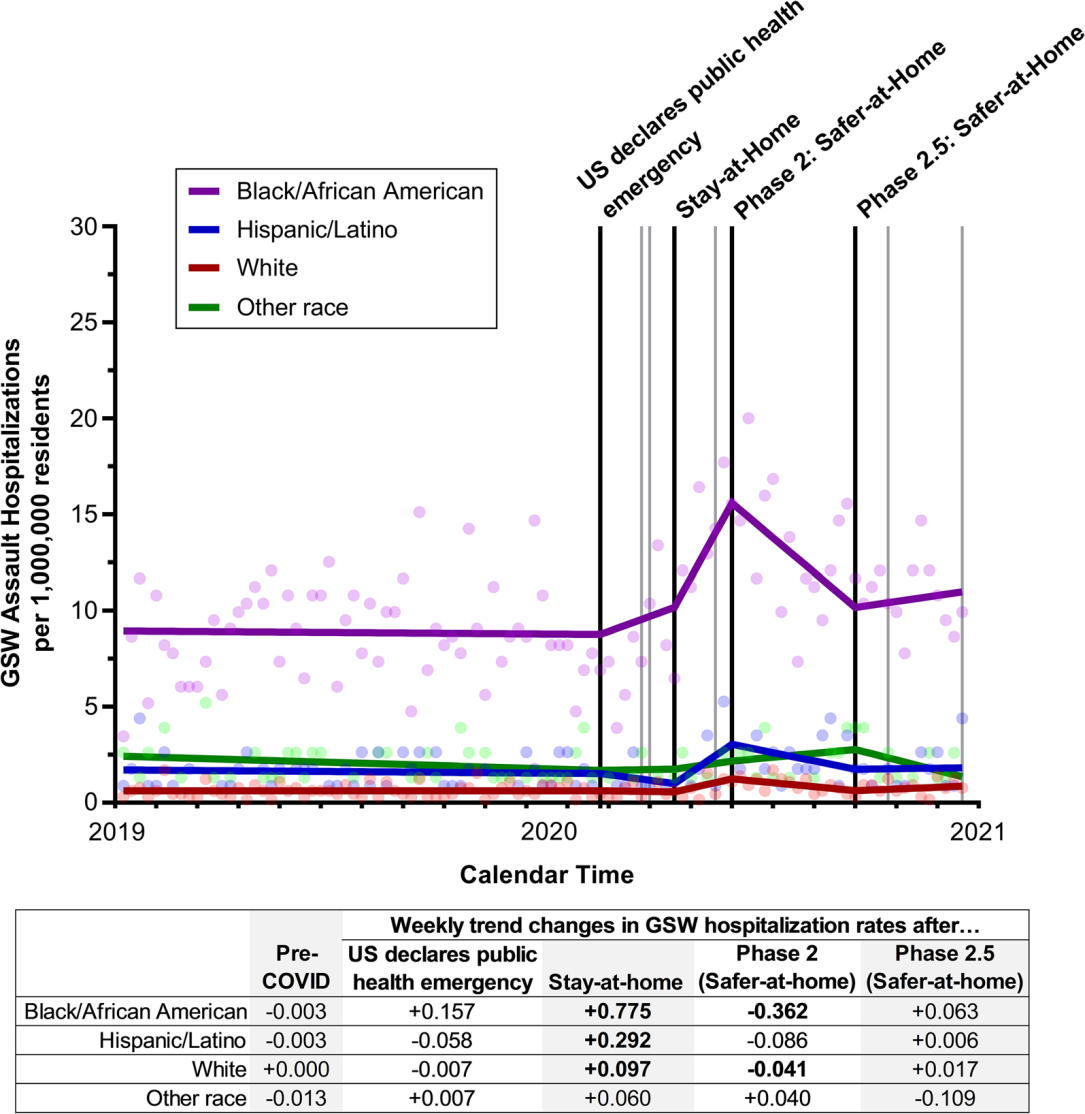
Weekly gunshot wound (GSW) assault hospitalizations per 1,000,000 North Carolina residents, 2019–2020, stratified by race/ethnicity. The black lines represent the timing of the four executive orders assessed in the analyses (U.S. declares public health emergency, North Carolina statewide Stay-at-Home order, statewide Phase 2: Safer-at-Home order, and statewide Phase 2.5: Safer-at-Home order); gray lines represent the time of the other COVID-related executive orders. Weekly trend changes in bold are statistically significant (*p* < 0.05)

Prior to the U.S. declaration of a public health emergency (January 2019–January 2020), substantial disparities in GSW hospitalization rates existed between Black/African American individuals and other racial/ethnic groups (average weekly rate: 8.9 per 1,000,000 vs. 0.6–2.1 per 1,000,000). After the Stay-at-Home order was implemented, hospitalization rates among Black/African American individuals began significantly increasing (weekly trend change = 0.775, 95% CI = 0.254 to 1.296, *p* = 0.004), peaking at an estimated 15.6 hospitalizations per 1,000,000 Black/African American residents by the time Phase 2: Safer-at-Home was enacted, Fig. 1 and Additional file 1: Table S5. After Stay-at-Home restrictions began to be lifted, GSW hospitalizations among Black/African American began decreasing (weekly trend change = − 0.362, 95% CI = − 0.618 to − 0.107, *p* = 0.007); however, by the end of 2020 weekly rates remained elevated compared to 2019 (average weekly hospitalization rate after Phase 2.5: Safer-at-Home = 10.6).

A smaller spike in GSW hospitalizations was also seen among Hispanic/Latino residents after Stay-at-Home orders were implemented (weekly trend change = 0.292, 95% CI = 0.018 to 0.566, *p* = 0.04), Fig. 1 and Additional file 1: Table S5. Weekly hospitalization rates among Hispanic/Latino residents began declining after restrictions were lifted, but the trend was not statistically significant (weekly trend change = − 0.086, 95% CI = -0.218 to 0.045, *p* = 0.20). The Stay-at-Home order also had a small impact on weekly GSW hospitalization rates among White residents (weekly trend change = 0.097, 95% CI = 0.024 to 0.170, *p* = 0.01), and no changes were seen among other race residents (weekly trend change = 0.060, 95% CI = − 0.166 to 0.286).

**Fig. 2:**
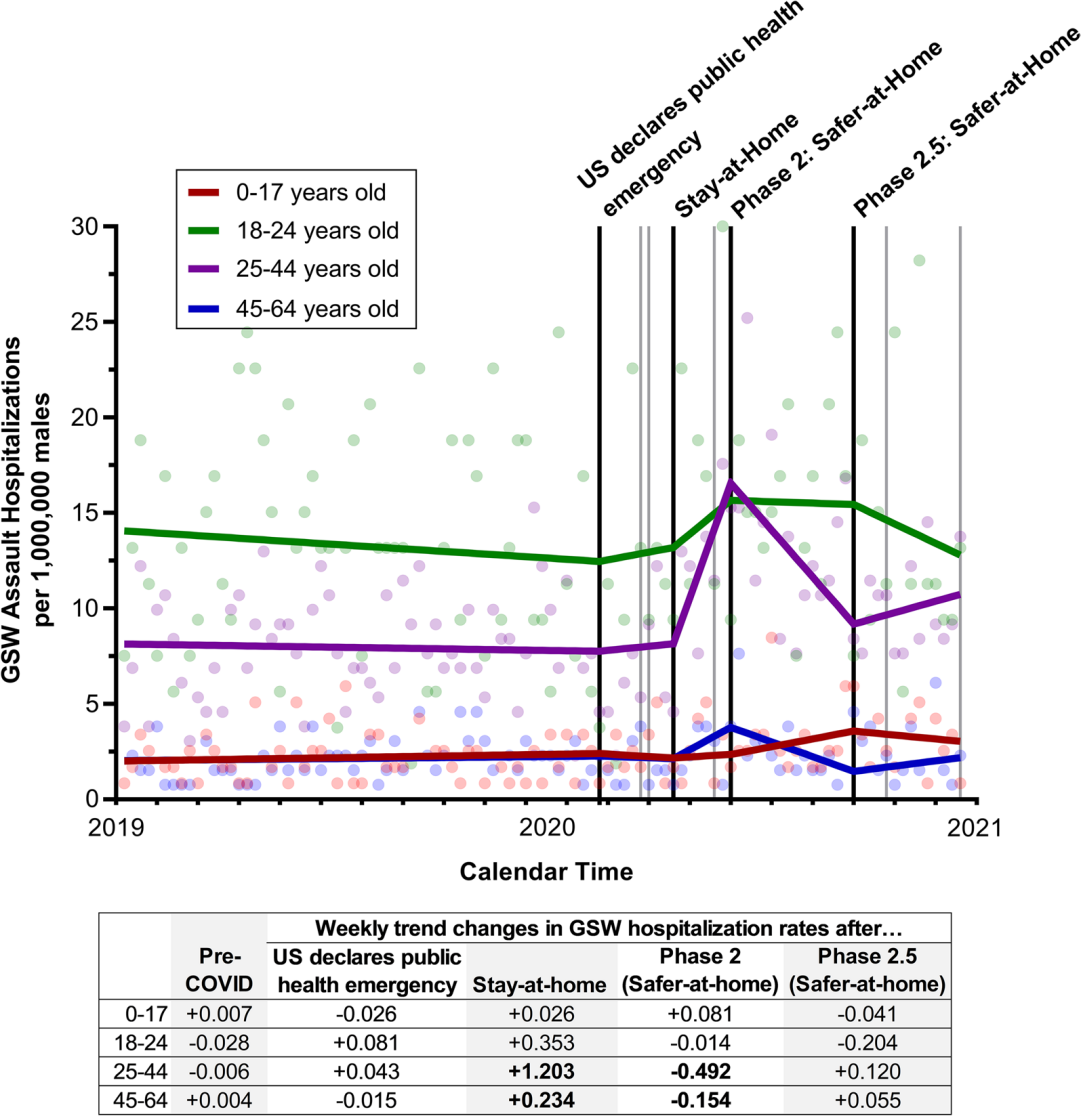
Weekly gunshot wound (GSW) assault hospitalizations per 1,000,000 North Carolina men, 2019–2020, stratified by age. The black lines represent the timing of the four executive orders assessed in the analyses (U.S. declares public health emergency, North Carolina statewide Stay-at-Home order, statewide Phase 2: Safer-at-Home order, and statewide Phase 2.5: Safer-at-Home order); gray lines represent the time of the other COVID-related executive orders. Older men (≥ 65 years old) were not included due to small numbers. Weekly trend changes in bold are statistically significant (*p* < 0.05)

Prior to the pandemic, GSW hospitalization rates were highest among 18–24-year-old males (average weekly rate = 13.3 hospitalizations), followed by 25–44-year-old males (average weekly rate = 7.9). After the Stay-at-Home order was implemented, GSW hospitalization rates began significantly increasing among 25–44-year-old males (weekly trend change = 1.202, 95% CI = 0.631 to 1.773, *p* < 0.0001), Fig. 2 and Additional file 1: Table S6. By the time restrictions began to be lifted (Phase 2: Safer-at-Home) 25–44-year-old males peaked at an estimated 15.7 GSW hospitalizations per week, similar to the estimated weekly GSW hospitalization rate among males 18–24 years old (estimated weekly rate = 16.6). By the end of 2020, GSW hospitalization rates remained elevated, compared to pre-pandemic levels, among males 25–44 years old (average weekly rate 10.1 vs. 7.9 per 1,000,000). A smaller increase and much lower GSW hospitalization rate was observed among males 45–64 years old (weekly trend change = 0.234, 95% CI = 0.008 to 0.461, *p* = 0.04). No statistically significant changes were observed among males 0–24 years old.

After the Stay-at-Home order, non-GSW hospitalization rates also began increasing among Black/African American residents (weekly trend change = 0.349, 95% CI = 0.027 to 0.671, *p* = 0.04) and males 25–44 years old (weekly trend change = 0.667, 95% CI = 0.149 to 1.185, *p* = 0.01), but increases were smaller than those observed among GSW hospitalization rates, Additional file 1: Fig. S1 and Tables S7–S8. By the end of 2020, non-GSW hospitalization rates were similar to pre-pandemic levels for both Black/African American residents (average weekly rate 6.8 vs. 7.1 pre-pandemic) and males 25–44 years old (average weekly rate 8.8 vs. 9.8 pre-pandemic).

## Discussion

Overall, in a statewide analysis of GSW assault hospitalizations before and during the first year of the COVID-19 pandemic, we found that GSW hospitalizations rates began dramatically increasing among Black/African American individuals and 25–44-year-old males after the Stay-at-Home order was enacted in North Carolina. Moreover, although rates decreased after restrictions were lifted between May and September 2020, GSW hospitalization rates remained elevated among both groups compared to pre-pandemic levels. By December 2020, there were still more than two additional GSW assault hospitalizations each week, per 1,000,000 residents, among both Black/African American individuals and males 25–44 years old. This trend was not observed among other racial/ethnic or age groups, or in non-GSW assault hospitalization rates. To the best of our knowledge, this is one of the first assessments of gun violence hospitalizations during the pandemic, and the first to look at the impact of specific COVID-related policies on GSW hospitalization rates, overall and across race/ethnicity and age.

Our results on gunshot wound hospitalizations closely mirror the disparities in increased firearm homicide rates in the U.S. reported by the CDC (Kegler et al. 2022) and others (Abdallah et al. 2021; Chen et al. 2020). Other U.S. states have also reported increased GSW hospitalization rates after Stay-at-Home orders (Yeates et al. 2021; Abdallah et al. 2021), but did not stratify by race/ethnicity or age, and only included data through June 2020. This analysis provides additional context and highlights that (1) gun violence hospitalizations among Black/African American individuals and men 25–44 years old have also increased during the pandemic, (2) these increases were associated with Stay-at-Home orders put in place to help combat the COVID-19 pandemic, and (3) while rates did decline after restrictions were lifted, they remained elevated compared to pre-pandemic levels. Taken together, these findings overwhelmingly highlight the need to reduce gun violence in the United States through effective and culturally tailored interventions and policies (Lee et al. 2017; Moyer et al. 2019; Bonne et al. 2021), including hospital-based programs (Brice and Boyle 2020).

The mechanisms causing increased GSW assault hospitalizations among Black/African American communities and adult men in North Carolina, and potentially other states, are likely complex. Potential causes include increased economic, social, and psychological hardships during the pandemic, especially during the early stages when statewide shutdowns of businesses and Stay-at-Home orders were in place. Research has suggested that Black/African American communities may have been impacted harder by the pandemic, compared to other racial/ethnic groups (Yancy 2020; Webb Hooper et al. 2020), which may explain why GSW rates increased more substantially among this group. Moreover, Black/African American communities have suffered also from cumulative long-term disadvantages due to structural racism and centuries of oppression, which also places them at higher risk of violence (Bailey et al. 2017). Black/African American individuals are also nearly three times more likely to be shot and killed by police as White Americans, further contributing to the gun violence in these communities (Edwards et al. 2018). Disruptions in employment, social services, and healthcare may also have played a role (Kegler et al. 2022). Given that the pandemic has continued to impact communities, even after restrictions were lifted, could explain why GSW hospitalization rates remain elevated, compared to pre-pandemic levels. Finally, given that no changes were seen among males 0–17 years old, it is possible that school closures did not adversely affect gun violence rates among school-aged children.

This study has a few limitations. First, while the NCTR includes all trauma hospitalizations across eighteen hospitals (including all trauma centers) in North Carolina, any injuries treated at non-participating sites, as well as individuals who died prior to reaching the hospital would not be included. Additionally, while we observed increases in GSW hospitalization rates after the Stay-at-Home order, there were several nationally recognized, co-occurring events and stressors that could have also led to these increases. We may also be limited in our ability to detect meaningful changes because several policies were implemented within a relatively short time, and several time periods had relatively few weekly data points.

## Conclusion

After a statewide Stay-at-Home order, gunshot wound hospitalizations increased substantially among Black/African American individuals and men 25–44 years old in North Carolina. While rates declined after restrictions were lifted, both groups saw sustained, increased GSW hospitalization rates for the rest of the year, compared to pre-pandemic rates. Increased rates after the Stay-at-Home order were also observed among Hispanic/Latino individuals. These results closely mirror previously reported increases in gunshot violence and homicides across the United States in 2020 but add critical context to the unequal burden experienced by racial/ethnic minorities and young men. Together, these findings highlight the need for comprehensive strategies aimed at reducing and preventing gun violence in the United States.

## Supplementary Information

**Additional file 1**. **Table S1** Dates and descriptions of COVID-19 executive orders in North Carolina. Bolded orders are ones included in analyses. **Table S2**. Demographics and clinical characteristics among GSW assault hospitalizations included in the North Carolina Trauma Registry, 2019-2020, stratified by race/ethnicity. **Table S3** Demographics and clinical characteristics among male GSW assault hospitalizations included in the North Carolina Trauma Registry, 2019-2020, stratified by age. Due to small counts (n=23), males ≥65 years old are not included. **Table S4** Demographics and clinical characteristics among all assault hospitalizations included in the North Carolina Trauma Registry, 2019-2020, stratified by year. **Table S5** Segmented linear regression modeling results for GSW assault hospitalization rates, stratified by race/ethnicity. **Table S6** Segmented linear regression modeling results for GSW assault hospitalization rates among males, stratified by age. **Table S7** Segmented linear regression modeling results for non-GSW assault hospitalization rates, stratified by race/ethnicity. **Table S8**. Segmented linear regression modeling results for non-GSW assault hospitalization rates among males, stratified by age. **Fig. S1** Weekly non-gunshot wound assault hospitalizations (e.g., struck with object) per 1,000,000 North Carolina residents, 2019-2020, stratified by A) race/ethnicity and B) age, among men only. The black lines represent the timing of the four executive orders assessed in the analyses (U.S. declares public health emergency, North Carolina statewide Stay-at-Home order, statewide Phase 2: Safer-at-Home order, and statewide Phase 2.5: Safer-at-Home order); gray lines represent the time of the other COVID-related executive orders. Weekly trend changes in bold are statistically significant (p<0.05).

## Data Availability

Data are available for request from the North Carolina Office of Emergency Medical Services. Researchers will make code available upon reasonable request. Please contact Dr. Paula Strassle for access.

## Abbreviations

CDC: Centers for Disease Control and Prevention
US: United States
GSW: Gunshot wound
NCTR: North Carolina Trauma Registry
IQR: Interquartile range
