# Supplementary material for: Impact of COVID-related policies on gunshot wound assault hospitalizations in the United States: a statewide time series analysis
Source: Inj Epidemiol. 2023 Jan 9;10:2. doi: 10.1186/s40621-022-00412-7 (PMC9829223; doi:10.1186/s40621-022-00412-7)

**Supplementary Table 1.** Dates and descriptions of COVID-19 executive orders in North Carolina. Bolded orders are ones included in analyses.

| **Order Date** | **Start Date** | **Description** |
| --- | --- | --- |
| **1/31/2020** | – | **US declares public health emergency** |
| 3/10/2020 | – | NC declares state of emergency |
| 3/13/2020 | – | US declares state of emergency |
| 3/14/2020 | 3/16/2020 | K-12 public schools closed statewide begins   - In effect until 3/30/2020 - Extended to 5/15 on 3/23/20 |
| **3/27/2020** | **3/30/2020**  **(5PM)** | **Statewide Stay-at-Home^a^ order issued**   - Extended to 5/8 on 4/23/20 |
| 5/5/2020 | 5/8/2020  (5PM) | Phase 1 (lessened some Stay-at-Home restrictions)   - In effect until 5/22/20 |
| **5/22/2020** | **5/22/2020**  **(5PM)** | **Phase 2 (Safer-at-Home)^b^ begins; Stay-at-Home order lifted**   - In effect until 6/26/20 - Extended to 7/17 on 6/24/20 - Extended to 8/7 on 7/16/20 - Extended to 9/11 on 8/5/20 |
| **9/1/2020** | **9/4/2020**  **(5PM)** | **Phase 2.5 (Safer-at-Home)^c^**   - In effect until 9/22/2020 |
| 9/30/2020 | 10/2/2020  (5PM) | Phase 3 (Safer-at-Home)^d^   - In effect until 10/23/20 - Extended to 11/13 on 10/21/20 - Extended to 12/4 on 11/10/20 |
| 12/8/2020 | 12/11/2020  (5PM) | Modified Stay-at-Home order^e^   - In effect until 1/8/21 |
| Reference: <https://www.nc.gov/covid-19/covid-19-orders#executive-orders>  ^a^ Stay-at-Home order required people to stay at home except for conducting essential businesses, engaging in outdoor exercise, or assisting family members. The order banned gatherings exceeding 10 people and stipulated people stay at least six feet apart from others  ^b^ Safer-at-Home (Phase 2) order reopened restaurants, childcare businesses, overnight camps, personal care facilities, indoor/outdoor pools, and sporting/entertainment venues that comply with public health requirements (e.g., six feet physical distancing, gatherings limited to 10 people indoors and 25 people outdoors)  ^c^ Safer-at-Home (Phase 2.5) order allowed gatherings to increase to 25 people indoors and 50 people outdoors, museums/aquariums and gyms to open at 50% and 30% capacity respectively, and playgrounds to become available. It also encouraged employers to provide employees with face masks  ^d^ Safer-at-Home (Phase 3) order allowed bars with outdoor seating, outdoor amusement parks, movie theaters, indoor meeting spaces, gaming facilities, and large outdoor facilities to reopen and comply with public health requirements (e.g., social distancing)  ^e^ Modified Stay-at-Home order stated that individuals remain in their place of stay and businesses and facilities close between 10:00pm and 5:00am | | |

**Supplementary Table 2.** Demographics and clinical characteristics among GSW assault hospitalizations included in the North Carolina Trauma Registry, 2019-2020, stratified by race/ethnicity.

|  | **Black/African American** | |  | **Hispanic/**  **Latino** | |  | **White** | | |  | **Other** | | |  |
| --- | --- | --- | --- | --- | --- | --- | --- | --- | --- | --- | --- | --- | --- | --- |
|  | **N** | **(%)** |  | **N** | **(%)** |  | **N** | **(%)** |  | | **N** | **(%)** |  |  |
| **Total, N** | 2,420 |  |  | 168 |  |  | 472 |  |  | | 107 |  |  |  |
| **Age, years, med (IQR)** | 26 | (20, 33) |  | 25 | (20, 34) |  | 31 | (23, 43) |  | | 27 | (20, 38) |  |  |
| **Age group, n (%)** |  |  |  |  |  |  |  |  |  | |  |  |  |  |
| 0-17 | 262 | (10.8) |  | 23 | (13.9) |  | 36 | (7.6) |  | | 13 | (12.1) |  |  |
| 18-24 | 793 | (32.8) |  | 55 | (33.1) |  | 108 | (22.9) |  | | 32 | (29.9) |  |  |
| 25-44 | 1,140 | (47.1) |  | 74 | (44.6) |  | 217 | (46.1) |  | | 45 | (42.1) |  |  |
| 45-64 | 204 | (8.4) |  | 14 | (8.4) |  | 100 | (21.2) |  | | 16 | (15.0) |  |  |
| ≥65 | 20 | (0.8) |  | 0 | (0.0) |  | 10 | (2.1) |  | | 1 | (0.9) |  |  |
| *Missing* | 1 |  |  | 2 |  |  | 1 |  |  | | 0 |  |  |  |
| **Male, n (%)** | 2,104 | (87.0) |  | 152 | (90.5) |  | 383 | (81.1) |  | | 86 | (80.4) |  |  |
| **Primary payer, n (%)** |  |  |  |  |  |  |  |  |  | |  |  |  |  |
| Any private insurance | 305 | (12.6) |  | 14 | (8.3) |  | 101 | (21.4) |  | | 16 | (15.0) |  |  |
| Medicare/Medicaid only | 704 | (29.2) |  | 42 | (25.0) |  | 119 | (25.3) |  | | 36 | (33.6) |  |  |
| Self-pay | 1,305 | (54.0) |  | 104 | (61.9) |  | 208 | (44.2) |  | | 50 | (46.7) |  |  |
| Other^a^ | 101 | (4.2) |  | 8 | (4.8) |  | 43 | (9.1) |  | | 5 | (4.7) |  |  |
| **Transferred to center, n (%)** | 607 | (26.9) |  | 33 | (22.0) |  | 98 | (23.3) |  | | 30 | (28.8) |  |  |
| **ISS, med (IQR)** | 9.0 | (2.0, 14.0) |  | 9.0 | (4.0, 16.0) |  | 9.0 | (2.0, 17.0) |  | | 10.0 | (5.0, 16.0) |  |  |
| **ED LOS, hours, med (IQR)** | 2.5 | (1.0, 4.4) |  | 2.7 | (1.1, 5.2) |  | 2.3 | (1.0, 3.9) |  | | 2.0 | (0.7, 4.2) |  |  |
| **LOS, days, med (IQR)** | 2.0 | (1.0, 6.0) |  | 2.0 | (1.0, 7.0) |  | 2.0 | (1.0, 6.0) |  | | 3.0 | (1.0, 8.0) |  |  |
| **ICU LOS^b^, days, med (IQR)** | 3.0 | (1.0, 5.0) |  | 2.0 | (1.0, 5.5) |  | 2.0 | (1.0, 5.0) |  | | 3.0 | (2.0, 5.0) |  |  |
| **Discharge disposition, n (%)** |  |  |  |  |  |  |  |  |  | |  |  |  |  |
| Routine/home | 1,945 | (83.6) |  | 137 | (85.6) |  | 348 | (78.9) |  | | 91 | (85.0) |  |  |
| Longterm care^c^ | 118 | (5.1) |  | 6 | (3.8) |  | 27 | (6.1) |  | | 2 | (1.9) |  |  |
| Transferred^d^ | 67 | (2.9) |  | 5 | (3.1) |  | 25 | (5.7) |  | | 3 | (2.8) |  |  |
| Died | 197 | (8.5) |  | 12 | (7.5) |  | 41 | (9.3) |  | | 11 | (10.3) |  |  |
| *Missing*^e^ | 93 |  |  | 8 |  |  | 31 |  |  | | 0 |  |  |  |
| **COVID-19 infection, n (%)** |  |  |  |  |  |  |  |  |  | |  |  |  |  |
| Confirmed | 20 | (0.8) |  | 1 | (0.6) |  | 1 | (0.2) |  | | 0 | (0.0) |  |  |
| Suspected | 181 | (7.5) |  | 17 | (10.1) |  | 34 | (7.2) |  | | 10 | (9.3) |  |  |
| Abbreviations: med, median; IQR, interquartile range; ISS, injury severity score; MVC, motor-vehicle collisions; ED, emergency department; LOS, length of stay; ICU, intensive care unit | | | | | | | | | | | | | | |
| ^a^ Other insurance types include worker’s compensation, other government insurance, Champus, and not billed | | | | | | | | | | | | | | |
| ^b^Among those admitted to ICU | | | | | | | | | | | | | | |
| ^c^ Long-term care includes: hospice, long-term care facility, nursing home, rehabilitation facility, skilled nursing facility (SNF) | | | | | | | | | | | | | | |
| ^d^Transfers to: acute care facilities, burn center, mental health facility, other trauma center, and transferred (unspecified) | | | | | | | | | | | | | | |
| ^e^ Includes individuals who left against medical advice | | | | | | | | | | | | | | |

**Supplementary Table 3.** Demographics and clinical characteristics among male GSW assault hospitalizations included in the North Carolina Trauma Registry, 2019-2020, stratified by age. Due to small counts (n=23), males ≥65 years old are not included.

|  | **0-17 years old** | |  | **18-24 years old** | |  | **25-44 years old** | |  | **45-64 years old** | |
| --- | --- | --- | --- | --- | --- | --- | --- | --- | --- | --- | --- |
|  | **N** | **(%)** |  | **N** | **(%)** |  | **N** | **(%)** |  | **N** | **(%)** |
| **Total, N** | 877 |  |  | 1,303 |  |  | 283 |  |  | 285 |  |
| **Race/ethnicity, n (%)** |  |  |  |  |  |  |  |  |  |  |  |
| Black/African American | 696 | (80.7) |  | 996 | (77.8) |  | 226 | (81.3) |  | 171 | (61.3) |
| Hispanic/Latino | 49 | (5.7) |  | 68 | (5.3) |  | 20 | (7.2) |  | 13 | (4.7) |
| White | 92 | (10.7) |  | 174 | (13.6) |  | 26 | (9.4) |  | 82 | (29.4) |
| Other | 25 | (2.9) |  | 42 | (3.3) |  | 6 | (2.2) |  | 13 | (4.7) |
| *Missing* | 15 |  |  | 23 |  |  | 5 |  |  | 6 |  |
| **Primary payer, n (%)** |  |  |  |  |  |  |  |  |  |  |  |
| Any private insurance | 156 | (17.8) |  | 135 | (10.4) |  | 28 | (9.9) |  | 48 | (16.8) |
| Medicare/Medicaid only | 234 | (26.8) |  | 183 | (14.1) |  | 213 | (75.3) |  | 66 | (23.2) |
| Self-pay | 442 | (50.6) |  | 910 | (70.0) |  | 37 | (13.1) |  | 153 | (53.7) |
| Other^a^ | 42 | (4.8) |  | 72 | (5.5) |  | 5 | (1.8) |  | 18 | (6.3) |
| **Transferred to center, n (%)** | 214 | (26.5) |  | 332 | (27.6) |  | 70 | (25.9) |  | 54 | (20.6) |
| **ISS, med (IQR)** | 9.0 | (2.0, 14.0) |  | 9.0 | (4.0, 16.0) |  | 9.0 | (1.0, 16.0) |  | 9.0 | (2.0, 14.0) |
| **ED LOS, hours, med (IQR)** | 2.6 | (1.1, 4.3) |  | 2.4 | (0.8, 4.3) |  | 2.1 | (0.8, 3.8) |  | 2.8 | (1.2, 4.5) |
| **LOS, days, med (IQR)** | 2.0 | (1.0, 5.0) |  | 2.0 | (1.0, 6.0) |  | 1.0 | (1.0, 6.0) |  | 2.0 | (1.0, 7.0) |
| **ICU LOS^b^, days, med (IQR)** | 2.0 | (1.0, 5.0) |  | 3.0 | (1.0, 5.0) |  | 3.0 | (2.0, 6.0) |  | 3.0 | (1.0, 6.0) |
| **Discharge disposition, n (%)** |  |  |  |  |  |  |  |  |  |  |  |
| Routine/home | 707 | (84.5) |  | 1,042 | (83.6) |  | 215 | (77.9) |  | 221 | (81.9) |
| Longterm care^c^ | 40 | (4.8) |  | 59 | (4.7) |  | 19 | (6.9) |  | 15 | (5.6) |
| Transferred^d^ | 21 | (2.5) |  | 31 | (2.5) |  | 16 | (5.8) |  | 15 | (5.6) |
| Died | 69 | (8.2) |  | 114 | (9.1) |  | 26 | (9.4) |  | 19 | (7.0) |
| *Missing*^e^ | 40 |  |  | 57 |  |  | 7 |  |  | 15 |  |
| **COVID-19 infection, n (%)** |  |  |  |  |  |  |  |  |  |  |  |
| Confirmed | 5 | (0.6) |  | 10 | (0.8) |  | 5 | (1.8) |  | 0 | (0.0) |
| Suspected | 57 | (6.5) |  | 109 | (8.4) |  | 16 | (5.7) |  | 18 | (6.3) |
| Abbreviations: med, median; IQR, interquartile range; ISS, injury severity score; MVC, motor-vehicle collisions; ED, emergency department; LOS, length of stay; ICU, intensive care unit | | | | | | | | | | | |
| ^a^ Other insurance types include worker’s compensation, other government insurance, Champus, and not billed | | | | | | | | | | | |
| ^b^Among those admitted to ICU | | | | | | | | | | | |
| ^c^ Long-term care includes: hospice, long-term care facility, nursing home, rehabilitation facility, skilled nursing facility (SNF) | | | | | | | | | | | |
| ^d^Transfers to: acute care facilities, burn center, mental health facility, other trauma center, and transferred (unspecified) | | | | | | | | | | | |
| ^e^ Includes individuals who left against medical advice | | | | | | | | | | | |

**Supplementary Table 4.** Demographics and clinical characteristics among all assault hospitalizations included in the North Carolina Trauma Registry, 2019-2020, stratified by year.

|  | **2019** | |  | **2020** | | **Standardized Difference^a^** |
| --- | --- | --- | --- | --- | --- | --- |
|  | **N** | **(%)** |  | **N** | **(%)** |  |
| **Total, N** | 3,253 |  |  | 3,565 |  | - |
| **Gunshot wound, n (%)** | 1,466 | (45.1) |  | 1,757 | (49.3) | 0.08 |
| **Age, years, med (IQR)** | 31 | (23, 43) |  | 31 | (23, 43) | 0.00 |
| **Age group, n (%)** |  |  |  |  |  |  |
| 0-17 | 219 | (6.7) |  | 262 | (7.4) | 0.02 |
| 18-24 | 750 | (23.1) |  | 752 | (21.1) | 0.05 |
| 25-44 | 1,527 | (47.0) |  | 1,728 | (48.5) | 0.03 |
| 45-64 | 656 | (20.2) |  | 710 | (19.9) | 0.01 |
| ≥65 | 99 | (3.0) |  | 108 | (3.0) | 0.00 |
| *Missing* | 2 |  |  | 5 |  | - |
| **Male, n (%)** | 2,693 | (82.8) |  | 2,951 | (82.9) | 0.00 |
| **Race/ethnicity, n (%)** |  |  |  |  |  |  |
| American Indian | 43 | (1.3) |  | 51 | (1.4) | 0.01 |
| Asian | 13 | (0.4) |  | 13 | (0.4) | 0.01 |
| Black/African American | 1,952 | (60.9) |  | 2,211 | (62.8) | 0.04 |
| Hispanic/Latino | 192 | (6.0) |  | 203 | (5.8) | 0.01 |
| White | 943 | (29.4) |  | 971 | (27.6) | 0.04 |
| Other^b^ | 48 | (1.5) |  | 53 | (1.5) | 0.00 |
| Multiracial | 15 | (0.5) |  | 20 | (0.6) | 0.00 |
| *Missing* | 47 |  |  | 43 |  | - |
| **Primary payer, n (%)** |  |  |  |  |  |  |
| Any private insurance | 479 | (14.7) |  | 478 | (13.4) | 0.04 |
| Medicare/Medicaid only | 873 | (26.9) |  | 1,068 | (30.0) | 0.07 |
| Self-pay | 1,666 | (51.3) |  | 1,742 | (48.9) | 0.05 |
| Other^c^ | 231 | (7.1) |  | 273 | (7.7) | 0.02 |
| **Transferred to center, n (%)** | 941 | (30.5) |  | 1,010 | (31.5) | 0.02 |
| **ISS, med (IQR)** | 5 | (1, 11) |  | 8 | (2, 13) | 0.08 |
| **ED LOS, hours, med (IQR)** | 3.3 | (1.7, 5.6) | | 3.3 | (1.5, 5.9) | 0.06 |
| **LOS, days, med (IQR)** | 1 | (1, 4) |  | 2 | (1, 4) | 0.06 |
| **ICU LOS^d^, days, med (IQR)** | 2 | (1, 4) |  | 2 | (1, 4) | 0.11 |
| **Discharge disposition, n (%)** |  |  |  |  |  |  |
| Routine/home | 2,708 | (87.0) |  | 2,920 | (86.5) | 0.02 |
| Longterm care^e^ | 135 | (4.3) |  | 142 | (4.2) | 0.02 |
| Transferred^f^ | 128 | (4.1) |  | 127 | (3.8) | 0.01 |
| Died | 140 | (4.5) |  | 188 | (5.6) | 0.05 |
| *Missing*^g^ | 142 |  |  | 188 |  | - |
| **COVID-19 infection, n (%)** |  |  |  |  |  |  |
| Confirmed | N/A |  |  | 40 | (1.1) | - |
| Suspected | N/A |  |  | 472 | (13.2) | - |
| Abbreviations: SD, standardized difference; med, median; IQR, interquartile range; ISS, injury severity score; MVC, motor-vehicle collisions; ED, emergency department; LOS, length of stay; ICU, intensive care unit | | | | | | |
| ^a^ Absolute standardized difference (SD) comparing demographics and clinical characteristics between 2019 and 2020; an SD >0.20 was considered meaningfully different | | | | | | |
| ^b^ Other race includes Other race and Hawaiian/Pacific Islander; race was collapsed due to small cell sizes | | | | | | |
| ^c^ Other insurance types include worker’s compensation, other government insurance, Champus, and not billed | | | | | | |
| ^d^Among those admitted to ICU | | | | | | |
| ^e^ Long-term care includes: hospice, long-term care facility, nursing home, rehabilitation facility, skilled nursing facility (SNF) | | | | | | |
| ^f^ Transfers to: acute care facilities, burn center, mental health facility, other trauma center, and transferred (unspecified) | | | | | | |
| ^g^ Includes individuals who left against medical advice | | | | | | |

**Supplementary Table 5.** Segmented Linear Regression Modeling Results for GSW assault hospitalization rates, stratified by race/ethnicity.

|  | **Black/African American** | | **Hispanic/Latino** | | **White** | | **Other race** | |
| --- | --- | --- | --- | --- | --- | --- | --- | --- |
|  | **Est.** | **(95% CI)** | **Est.** | **(95% CI)** | **Est.** | **(95% CI)** | **Est.** | **(95% CI)** |
| **Intercept** | 8.950 | (7.424, 10.475) | 1.710 | (1.017, 2.403) | 0.623 | (0.412, 0.834) | 2.437 | (1.738, 3.136) |
| **Pre-COVID Trend Change** | -0.003 | (-0.047, 0.041) | -0.003 | (-0.023, 0.017) | 0.000 | (-0.006, 0.006) | -0.013 | (-0.033, 0.007) |
| **Trend Change after Declaration^a^** | 0.160 | (-0.262, 0.581) | -0.055 | (-0.282, 0.171) | -0.007 | (-0.065, 0.052) | 0.020 | (-0.158, 0.198) |
| **Trend Change after Stay-at-Home** | 0.618 | (-0.211, 1.448) | 0.351 | (-0.100, 0.801) | 0.104 | (-0.013, 0.220) | 0.053 | (-0.301, 0.407) |
| **Trend Change after Phase 2: Safer-at-Home** | -1.138 | (-1.809, -0.466) | -0.379 | (-0.721. -0.036) | -0.138 | (-0.231, -0.045) | -0.020 | (-0.308, 0.268) |
| **Trend Change after Phase 2.5: Safer at Home** | 0.425 | (-0.056, 0.906) | 0.092 | (-0.147, 0.331) | 0.058 | (-0.007, 0.124) | -0.149 | (-0.352, 0.054) |
| Note: The intercept represents the average rate of hospitalizations, per 1,000,000, at baseline (Jan 2019). To calculate the slope between two time points, add the trend after the time point of interest with all prior weekly trend changes. For example, to estimated slope for the rate of GSW assault hospitalizations among Black/African American residents after the Stay-at-Home order was issued is calculated as (-0.003) + (0.160) + (0.618) = 0.775 and interpreted as an average weekly increase of 0.775 GSW hospitalizations, per 1,000,000 Black/African American residents, after the Stay-at-Home order, but before the Phase 2: Safer-at-Home order  Abbreviations: Est, estimate; CI, confidence interval  ^a^ Trend after U.S. declaration of a public health emergency | | | | | | | | |

**Supplementary Table 6.** Segmented Linear Regression Modeling Results for GSW assault hospitalization rates among males, stratified by age.

|  | **0-17 years old** | | **18-24 years old** | | **25-44 years old** | | **45-64 years old** | |
| --- | --- | --- | --- | --- | --- | --- | --- | --- |
|  | **Est.** | **(95% CI)** | **Est.** | **(95% CI)** | **Est.** | **(95% CI)** | **Est.** | **(95% CI)** |
| **Intercept** | 2.005 | (1.275, 2.736) | 14.086 | (10.782, 17.391) | 8.141 | (6.470, 9.811) | 2.027 | (1.330, 2.724) |
| **Pre-COVID Trend Change** | 0.007 | (-0.013, 0.027) | -0.028 | (-0.121, 0.066) | -0.006 | (-0.055, 0.042) | 0.004 | (-0.015, 0.024) |
| **Trend Change after Declaration^a^** | -0.032 | (-0.231, 0.167) | 0.108 | (-0.806, 1.023) | 0.050 | (-0.414, 0.513) | -0.019 | (-0.204, 0.166) |
| **Trend Change after Stay-at-Home** | 0.052 | (-0.355, 0.459) | 0.272 | (-1.572, 2.116) | 1.159 | (0.251, 2.067) | 0.249 | (-0.115, 0.613) |
| **Trend Change after Phase 2: Safer-at-Home** | 0.055 | (-0.276, 0.387) | -0.367 | (-1.864, 1.130) | -1.694 | (-2.428, -0.960) | -0.388 | (-0.681, -0.095) |
| **Trend Change after Phase 2.5: Safer at Home** | -0.123 | (-0.361, 0.115) | -0.189 | (-1.257, 0.879) | 0.612 | (0.087, 1.137) | 0.209 | (-0.010, 0.428) |
| Note: The intercept represents the average rate of hospitalizations, per 1,000,000, at baseline (Jan 2019). To calculate the slope between two time points, add the trend after the time point of interest with all prior weekly trend changes. For example, to estimated slope for the rate of GSW assault hospitalizations among Black/African American residents after the Stay-at-Home order was issued is calculated as (-0.006) + (0.050) + (1.159) = 1.203 and interpreted as an average weekly increase of 1.203 GSW hospitalizations, per 1,000,000, after the Stay-at-Home order, but before the Phase 2: Safer-at-Home order  Abbreviations: Est, estimate; CI, confidence interval  ^a^ Trend after U.S. declaration of a public health emergency | | | | | | | | |

**Supplementary Table 7.** Segmented Linear Regression Modeling Results for non-GSW assault hospitalization rates, stratified by race/ethnicity.

|  | **Black/African American** | | **Hispanic/Latino** | | **White** | | **Other race** | |
| --- | --- | --- | --- | --- | --- | --- | --- | --- |
|  | **Est.** | **(95% CI)** | **Est.** | **(95% CI)** | **Est.** | **(95% CI)** | **Est.** | **(95% CI)** |
| **Intercept** | 6.682 | (5.728, 7.636) | 2.468 | (1.618, 3.318) | 2.200 | (1.899, 2.501) | 1.812 | (1.290, 2.334) |
| **Pre-COVID Trend Change** | 0.015 | (-0.013, 0.043) | 0.001 | (-0.023, 0.026) | -0.001 | (-0.010, 0.007) | 0.001 | (-0.014, 0.016) |
| **Trend Change after Declaration^a^** | -0.144 | (-0.410, 0.122) | -0.055 | (-0.262, 0.151) | -0.033 | (-0.116, 0.050) | -0.037 | (-0.191, 0.118) |
| **Trend Change after Stay-at-Home** | 0.478 | (-0.035, 0.991) | 0.051 | (-0.332, 0.434) | 0.189 | (0.021, 0.357) | 0.168 | (-0.141, 0.478) |
| **Trend Change after Phase 2: Safer-at-Home** | -0.466 | (-0.878, -0.053) | 0.041 | (-0.264, 0.346) | -0.235 | (-0.371, -0..098) | -0.176 | (-0.430, 0.079) |
| **Trend Change after Phase 2.5: Safer at Home** | 0.080 | (-0.217, 0.378) | -0.214 | (-0.467, 0.039) | 0.089 | (-0.008, 0.186) | 0.170 | (-0.019, 0.359) |
| Note: The intercept represents the average rate of hospitalizations, per 1,000,000, at baseline (Jan 2019). To calculate the slope between two time points, add the trend after the time point of interest with all prior weekly trend changes. For example, to estimated slope for the rate of non-GSW assault hospitalizations among Black/African American residents after the Stay-at-Home order was issued is calculated as (0.015) + (-0.144) + (0.478) = 0.349 and interpreted as an average weekly increase of 0.349 non-GSW hospitalizations, per 1,000,000, after the Stay-at-Home order, but before the Phase 2: Safer-at-Home order  Abbreviations: Est, estimate; CI, confidence interval  ^a^ Trend after U.S. declaration of a public health emergency | | | | | | | | |

**Supplementary Table 8.** Segmented Linear Regression Modeling Results for non-GSW assault hospitalization rates among males, stratified by age.

|  | **0-17 years old** | | **18-24 years old** | | **25-44 years old** | | **45-64 years old** | |
| --- | --- | --- | --- | --- | --- | --- | --- | --- |
|  | **Est.** | **(95% CI)** | **Est.** | **(95% CI)** | **Est.** | **(95% CI)** | **Est.** | **(95% CI)** |
| **Intercept** | 1.184 | (0.898, 1.469) | 7.005 | (5.298, 8.713) | 9.209 | (7.734, 10.684) | 5.753 | (4.460, 7.047) |
| **Pre-COVID Trend Change** | 0.001 | (-0.007, 0.010) | -0.012 | (-0.062, 0.038) | 0.021 | (-0.021, 0.063) | 0.018 | (-0.019, 0.055) |
| **Trend Change after Declaration^a^** | 0.014 | (-0.069, 0.097) | 0.108 | (-0.355, 0.572) | -0.306 | (-0.714, 0.102) | -0.177 | (-0.536, 0.181) |
| **Trend Change after Stay-at-Home** | -0.097 | (-0.260, 0.066) | 0.076 | (-0.820, 0.973) | 0.953 | (0.129, 1.777) | 0.505 | (-0.202, 1.212) |
| **Trend Change after Phase 2: Safer-at-Home** | 0.097 | (-0.041, 0.234) | -0.562 | (-1.300, 0.175) | -0.892 | (-1.562, -0.222) | -0.434 | (-1.003, 0.135) |
| **Trend Change after Phase 2.5: Safer at Home** | 0.033 | (-0.100, 0.167) | 0.611 | (0.065, 1.158) | 0.181 | (-0.293, 0.655) | 0.050 | (-0.355, 0.454) |
| Note: The intercept represents the average rate of hospitalizations, per 1,000,000, at baseline (Jan 2019). To calculate the slope between two time points, add the trend after the time point of interest with all prior weekly trend changes. For example, to estimated slope for the rate of non-GSW assault hospitalizations among males 25-44 years old after the Stay-at-Home order was issued is calculated as (0.021) + (-0.306) + (0.953) = 0.668 and interpreted as an average weekly increase of 0.668 assault GSW hospitalizations, per 1,000,000 men aged 25-44 years old, after the Stay-at-Home order, but before the Phase 2: Safer-at-Home order  Abbreviations: Est, estimate; CI, confidence interval  ^a^ Trend after U.S. declaration of a public health emergency | | | | | | | | |

**Supplementary Figure 1.** Weekly non-gunshot wound assault hospitalizations (e.g., struck with object) per 1,000,000 North Carolina residents, 2019-2020, stratified by A) race/ethnicity and B) age, among men only. The black lines represent the timing of the four executive orders assessed in the analyses (U.S. declares public health emergency, North Carolina statewide Stay-at-Home order, statewide Phase 2: Safer-at-Home order, and statewide Phase 2.5: Safer-at-Home order); grey lines represent the time of the other COVID-related executive orders. Weekly trend changes in **bold** are statistically significant (p<0.05).


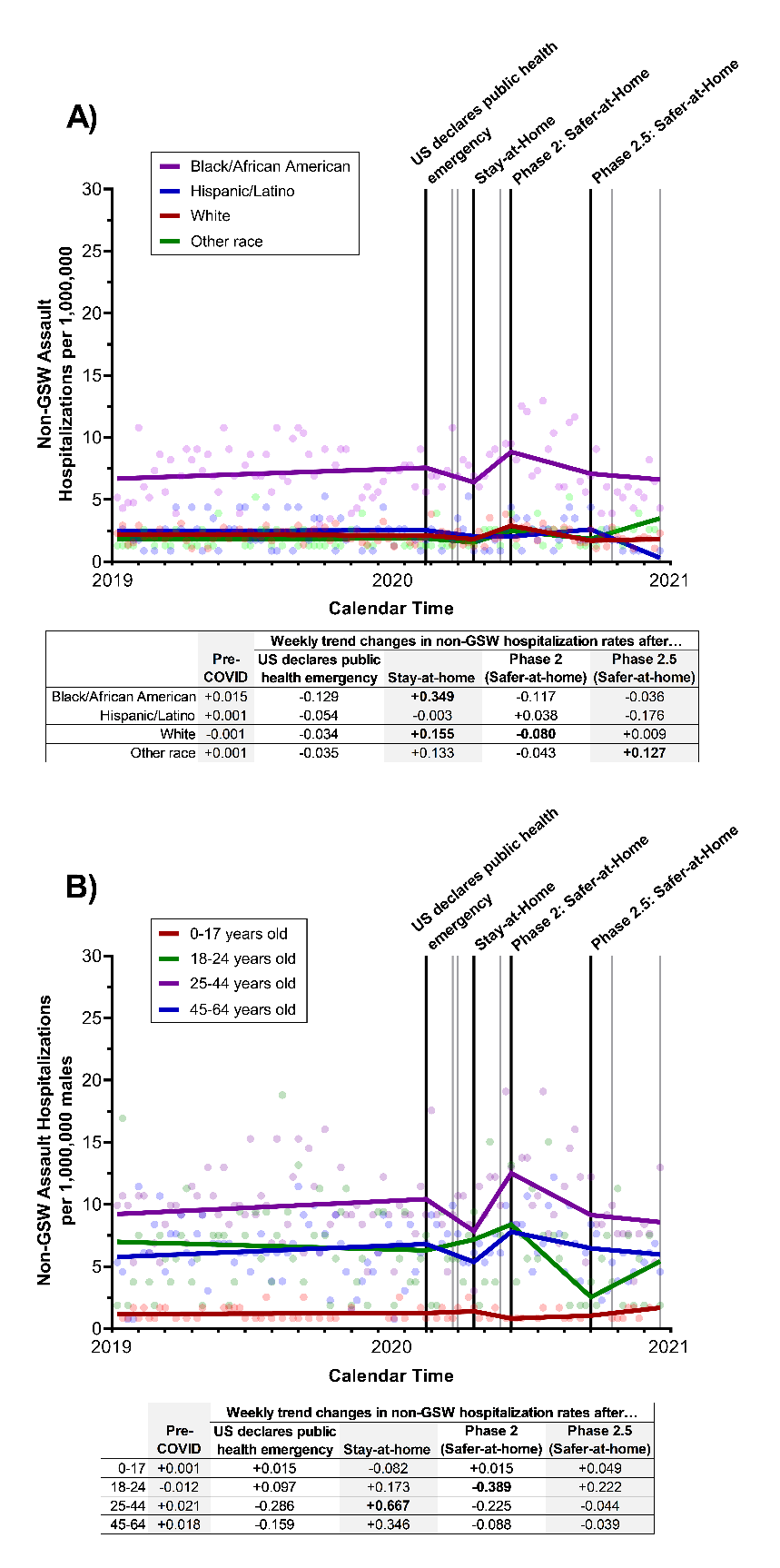

Supplement: Supplementary file 1 — Additional file 1. Table S1 Dates and descriptions of COVID-19 executive orders in North Carolina. Bolded orders are ones included in analyses. Table S2. Demographics and clinical characteristics among GSW assault hospitalizations included in the North Carolina Trauma Registry, 2019-2020, stratified by race/ethnicity. Table S3 Demographics and clinical characteristics among male GSW assault hospitalizations included in the North Carolina Trauma Registry, 2019-2020, stratified by age. Due to small counts (n=23), males ≥65 years old are not included. Table S4 Demographics and clinical characteristics among all assault hospitalizations included in the North Carolina Trauma Registry, 2019-2020, stratified by year. Table S5 Segmented linear regression modeling results for GSW assault hospitalization rates, stratified by race/ethnicity. Table S6 Segmented linear regression modeling results for GSW assault hospitalization rates among males, stratified by age. Table S7 Segmented linear regression modeling results for non-GSW assault hospitalization rates, stratified by race/ethnicity. Table S8. Segmented linear regression modeling results for non-GSW assault hospitalization rates among males, stratified by age. Fig. S1 Weekly non-gunshot wound assault hospitalizations (e.g., struck with object) per 1,000,000 North Carolina residents, 2019-2020, stratified by A) race/ethnicity and B) age, among men only. The black lines represent the timing of the four executive orders assessed in the analyses (U.S. declares public health emergency, North Carolina statewide Stay-at-Home order, statewide Phase 2: Safer-at-Home order, and statewide Phase 2.5: Safer-at-Home order); gray lines represent the time of the other COVID-related executive orders. Weekly trend changes in bold are statistically significant (p<0.05). [file 40621_2022_412_MOESM1_ESM.docx]
